# Attenuating MKRN1 E3 ligase-mediated AMPKα suppression increases tolerance against metabolic stresses in mice

**DOI:** 10.15698/cst2018.11.164

**Published:** 2018-10-11

**Authors:** Hyunji Han, Sehyun Chae, Daehee Hwang, Jaewhan Song

**Affiliations:** 1 Department of Biochemistry, College of Life Science and Biotechnology, Yonsei University, Seoul 03722, Republic of Korea.; 2 Center for Plant Aging Research, Institute for Basic Science.; 3 Department of New Biology, Daegu Gyeongbuk Institute of Science and Technology (DGIST), Daegu 42988, Republic of Korea.

**Keywords:** AMPK, MKRN1, metabolic syndrome, obesity, diabetes

## Abstract

The 5' adenosine monophosphate-activated protein kinase (AMPK) is an essential energy sensor in the cell, which, at low energy levels, instigates the cellular energy-generating systems along with suppression of the anabolic signaling pathways. The activation of AMPK through phosphorylation is a well-known process; however, activation alone is not sufficient, and knowledge about the other regulatory networks of post-translational modifications connecting the activities of AMPK to systemic metabolic syndromes is important, which is still lacking. The recent studies on Makorin Ring Finger Protein 1 (MKRN1) mediating the ubiquitination and proteasome-dependent degradation of AMPK( implicate that the post-translational modification of AMPK, regulating its protein homeostasis, could impose significant systemic metabolic effects (Lee *et al*. Nat Commun 9:3404). In this study, MKRN1 was identified as a novel E3 ligase for both AMPKα1 and α2. Mouse embryonic fibroblasts, genetically deleted for *Mkrmn1*, and *Ampkα1* and *α2*, became stabilized with the suppression of lipogenesis pathways and an increase in nutrient consumption and mitochondria regeneration. Of note, the *Mkrn1* knockout mice fed normal chow displayed no obvious phenotypic defects or abnormality, whereas the *Mkrn1*-null mice exhibited strong tolerance to metabolic stresses induced by high-fat diet (HFD). Thus, these mice, when compared with the HFD-induced wild type, were resistant to obesity, diabetes, and non-alcoholic fatty liver disease. Interestingly, in whole-body *Mkrn1* knockout mouse, only the liver and white and brown adipose tissues displayed anincrease in the active phosphorylated AMPK levels, but no other organs, such as the hypothalamus, skeletal muscles, or pancreas, displayed such increases. Specific ablation of MKRN1 in the mouse liver using adenovirus prevented HFD-induced lipid accumulation in the liver and blood, implicating MKRN1 as a possible therapeutic target for metabolic syndromes, such as obesity, type II diabetes, and fat liver diseases. This study would provide a crucial perspective on the importance of post-translational regulation of AMPK in metabolic pathways and will help researchers develop novel therapeutic strategies that target not only AMPK but also its regulators.

The signaling pathways instigated by the activation of AMP-activated protein kinase (AMPK) play major roles in balancing the energy levels in cells as well as in the whole-body system by stimulating the energy expenditure. Recent studies have expanded the roles of AMPK in a variety of metabolic syndromes, such as obesity, type 2 diabetes, fatty liver syndrome, cardiovascular diseases, and tumorigenesis. In particular, its effects on the formation of brown and beige adipose tissues and regeneration of mitochondria make AMPK an attractive target to control or cure metabolic diseases. As a cellular energy-sensing enzyme, AMPK exists as diverse heterotrimeric complexes comprised of a catalytic (α1 or α2) subunit and two regulatory subunits (β1 or β2 and γ1, γ2 or γ3). Under the energy stress condition of higher AMP/ATP ratio, AMP binds to γ subunits, allosterically activating AMPK, leading to an induction of catabolism with concomitant suppression of anabolism. While the phosphorylation of AMPK has been unveiled to be mediated by kinases such as liver kinase B1 (LKB1) and calcium calmodulin-dependent protein kinase kinase β (CAMKKβ), regulation of AMPK by other post-translational modifications, such as ubiquitination, still need to be elucidated. This regulatory mechanism is necessary to understand the relevant physiological effects of AMPK, including metabolic syndromes. In the recent studies of E3 ligase, MKRN1, and AMPKα, it has been shown that the suppression of AMPKα activities by MKRN1 could have metabolically systemic effects. Of note, while MKRN1 is expressed in most of the organs, the *Mkrn1* knockout mouse shows an increase of levels of AMPK and its activities only in the liver and adipose tissues, the two major metabolic organs. Accordingly, lipid accumulation in this knockout mouse was prevented as expected for mice with higher AMPK levels. This leads to the prevention of diseases related to metabolic syndromes, such as lowerblood lipid and cholesterol levels, insulin intolerance, type II diabetes, and steatosis. Since MKRN1 is generally expressed in most organs, it is of interest to identify a factor that might suppress MRKN1 and AMPKα interaction in organs other than the liver and adipose tissues. The effects of *Mkrn1* knockout on AMPK activation *in vivo* in the presence of HFD were comprehensively interpreted by cellular pathways represented by the differentially expressed genes (DEGs) between *Mkrn1* knockout and wild type mice in the liver and adipose tissues. In **Figure 1**, the effects of *Mkrn1* depletion on AMPK activation in the mouse liver and adipose tissues are presented based on the pathways suppressed or activated. These analyses showed that the pathways associated with lipid consumption or accumulation were the most affected by *Mkrn1* knockout. As expected, enzymes such as ACACA/B, FASN, TECR, HSD17B12, ELOVL1/2/5/6/7, SCD1/2, and ACOT, which are related to lipid anabolism starting from acetyl-CoA, were considerably suppressed upon *Mkrn1* depletion. On the other hand, the pathways involved in lipolysis were up-regulated, corroborating the effect of active AMPK. Notably, the genes including *Pgc1(*, *Cidea*, *Prdm16*, and *Ucp1*, which are involved in the differentiation and function of brown adipocytes, were up-regulated. Although we did not pursue the differentiation of brown or beige adipocytes under various environmental conditions in this study, the up-regulation of these genes warrants further investigations to elucidate the possible association of *Mkrn1* depletion in facilitating the browning or beiging process of adipocytes. In the *Mkrn1 *knockout mice, glucose uptake and consumption were generally up-regulated in the adipose tissues, while gluconeogenesis was down-regulated in the liver, which corroborates the diabetes-free condition in *Mkrn1* knockout mice fed HFD. Interestingly, in *Mkrn1* knockout mice, the upstream and downstream pathways of mTOR were globally suppressed in the liver. For example, the AKT pathway was down-regulated compared to that in the wild type mice fed HFD. In corroboration with these data, the downstream targets of mTOR were also inhibited, indicating that activation of AMPK upon suppression of MKRN1 would negatively regulate the mTOR pathway. The extent of inhibition of mTOR upon *Mkrn1* knockout, however, might involve other factors regulated by MKRN1.

**Figure 1:**
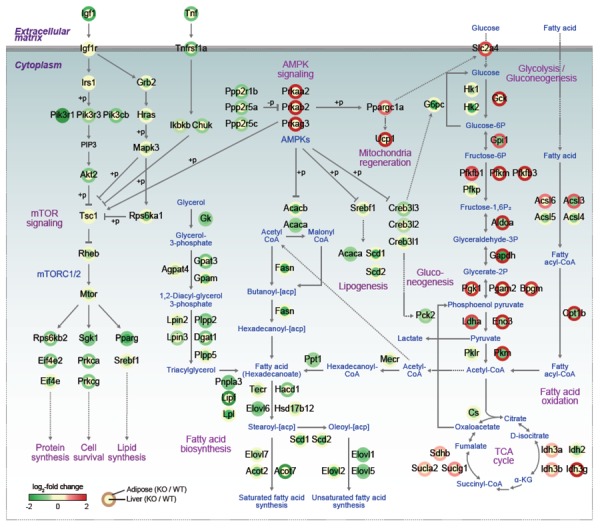
FIGURE 1: Regulation of Metabolism in MKRN1 knockout mice. Network models describing alterations of cellular processes regulated by DEGs in MKRN1-null liver and adipose tissue. Solid lines denote metabolic reactions and dotted lines denote transports of molecules or transcriptional regulations between transcriptional regulators and their target genes. Node center and border colors represent up- (red) or down-regulation (green) in MKRN1-null liver (center) and adipose tissue (border; see the legend for node center and border). The color bar represents the gradient of log_2_-fold-changes of mRNA expression levels by MKRN1 ablation relative to those in WT. Plasma membranes were denoted by thick gray lines, cytoplasm by blue background, and extracellular matrix by white background.

While the influence of MKRN1 on AMPK seems to have a very potent effect on the regulation of metabolic pathways, the other signaling pathways regulated by MKRN1 could not be ignored. MKRN1 is an E3 ligase engaged in various signaling pathways. In particular, it is known to target potent tumor suppressors closely associated with p53. MKRN1 degrades p14ARF, which is induced by an oncogene, such as c-Myc, and also is a negative regulator of MDM2, an obligatory E3 ligase of p53 (**Figure 2**). Thus, oncogenic signaling instigates the senescence process by activating p14ARF and subsequently p53 as a way to prevent cells from becoming tumorigenic. The tumor-suppressor protein p53 is also a transcriptional activator of PTEN, a phosphatase that dephosphorylates phosphatidylinositol-3,4,5-trisphosphate to phosphatidylinositol-4,5-trisphosphate. This process suppresses the downstream signaling of phosphatidylinositide 3 (PI3)-kinase, which leads to the inactivation of AKT and subsequently mTOR, which is the master regulator of anabolic pathways. Thus, activation of p53 could result in cellular senescence with suppression of anabolic processes, a typical signature preventing an oncogenic process. Of note, MKRN1 could induce the degradation of p14ARF, p53, and PTEN, which could lead to the rearrangement of metabolic homeostasis causing possible induction or suppression of anabolism or catabolism, respectively. In addition to this plausible tendency of MKRN1 to promote anabolism, MKRN1 is also capable to induce the ubiquitination and proteasome-dependent degradation of AMPK(, which would potentiate these processes. It would be interesting to further investigate the effects of MKRN1-mediated AMPK suppression on p53 and mTOR, the targets of AMPK. Based on these observations, MKRN1 could be considered as a regulatory factor that inhibits the cellular energy consumption process, thus prompting the accumulation of metabolic macromolecules. Besides these functions, MKRN1 also acts as a negative regulator of FADD (Fas-associated death domain) and APC (Adenomatous polyposis coli) proteins, which are essential factors in cytokine-dependent cell death and Wnt signaling. An association of these pathways with the metabolic processes, involving the AMPK pathway regulated by MKRN1, could not be excluded as a plausible crosstalk.

**Figure 2:**
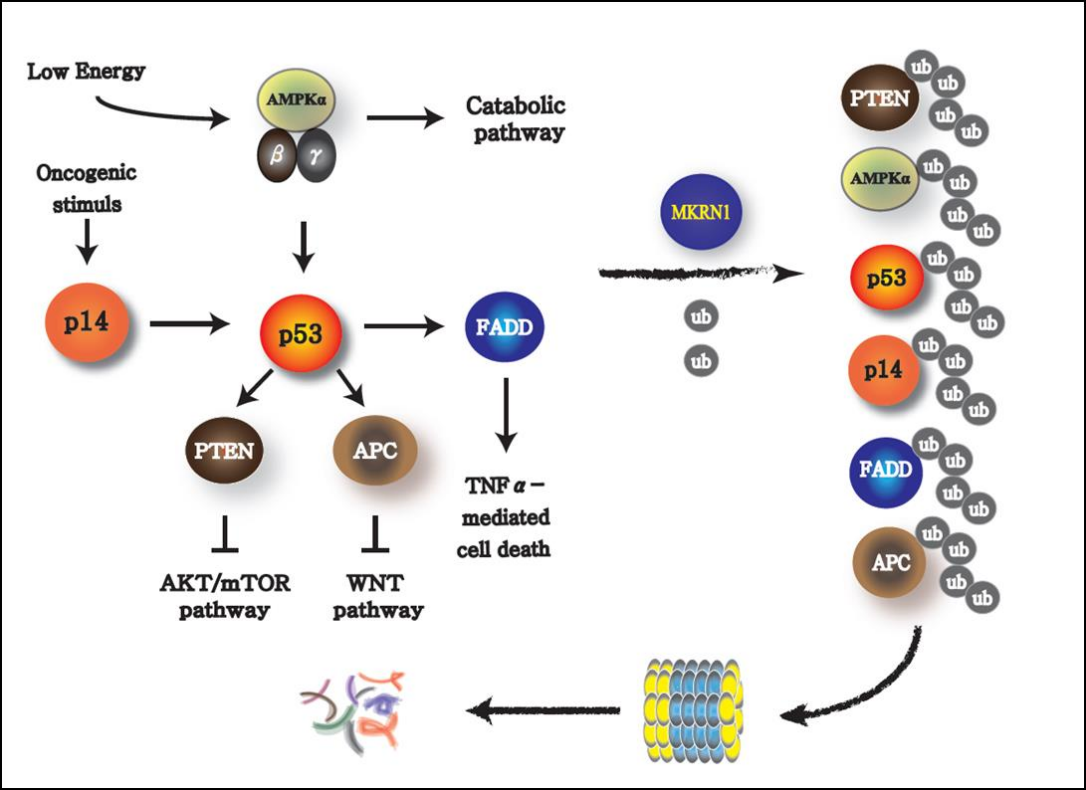
FIGURE 2: Targets of MKRN1. MKRN1 mediates the ubiquitination of AMPKα, p14ARF, p53, FADD, PTEN, and APC, which are then degraded via the proteasome-dependent pathway. AMPK, the energy sensor inducing catabolism, is also an activator of p53, which in turn enforces the function of PTEN, APC, and FADD via direct or indirect regulation. In particular, induction of PTEN by p53 leads to the suppression of AKT and thus of mTOR pathways, inhibiting the anabolic pathways. Thus, MKRN1 might have comprehensive effects on general metabolic pathways by regulating the factors involved.

In summary, MKRN1 functions as an E3 ubiquitin ligase prompting AMPKα destabilization, specifically in the liver and white and brown adipose tissues. AMPK activation under depletion of *Mkrn1* in mice accelerated the consumption of nutrients, including lipids, in these tissues, facilitating more energy expenditure and preventing metabolic syndromes, such as the liver steatosis, insulin resistance, and obesity induced by HFD. The therapeutic potential of MKRN1 inhibition in hyperglycemia and hepatic steatosis was revealed using adenoviral gene specifically delivered to the liver. Further studies using a conditional *Mkrn1* knockout mouse are required to investigate the protein’s therapeutic effects on serious metabolic diseases, such as nonalcoholic steatohepatitis.

